# FragMAX: the fragment-screening platform at the MAX IV Laboratory

**DOI:** 10.1107/S205979832000889X

**Published:** 2020-07-27

**Authors:** Gustavo M. A. Lima, Vladimir O. Talibov, Elmir Jagudin, Céleste Sele, Maria Nyblom, Wolfgang Knecht, Derek T. Logan, Tove Sjögren, Uwe Mueller

**Affiliations:** a BioMAX, MAX IV Laboratory, Fotongatan 2, 224 84 Lund, Sweden; b Department of Biology and Lund Protein Production Platform, Lund University, Sölvegatan 35, 22362 Lund, Sweden; c Biochemistry and Structural Biology, Department of Chemistry, Lund University, Box 124, 221 00 Lund, Sweden; d SARomics Biostructures AB, Medicon Village, Scheeletorget 1, 223 63 Lund, Sweden; e Discovery Sciences, BioPharmaceuticals R&D, AstraZeneca, 431 83 Mölndal, Sweden; f Macromolecular Crystallography, Helmholtz-Zentrum Berlin, Albert-Einstein-Strasse 15, 12489 Berlin, Germany

**Keywords:** fragment screening, high-throughput data analysis, protein crystallography, drug discovery, software, user facility, BioMAX, FragMAX

## Abstract

Advances in synchrotron storage rings and beamline automation have pushed data-collection rates to thousands of data sets per week. With this increase in throughput, massive projects such as in-crystal fragment screening have become accessible to a larger number of research groups. The quality of support offered at large-scale facilities allows medicinal chemistry-focused or biochemistry-focused groups to supplement their research with structural biology. Preparing the experiment, analysing multiple data sets and prospecting for interesting complexes of protein and fragments require, for both newcomers and experienced users, efficient management of the project and extensive computational power for data processing and structure refinement. Here, FragMAX, a new complete platform for fragment screening at the BioMAX beamline of the MAX IV Laboratory, is described. The ways in which users are assisted in X-ray-based fragment screenings and in which the fourth-generation storage ring available at the facility is best exploited are also described.

## Abstract 2

FragMAX is the fragment-screening platform of BioMAX, the macromolecular crystallography beamline of the MAX IV Laboratory. It provides support for users during sample preparation, data collection and data analysis through its in-house-developed software, *FragMAXapp*.

## 1. Introduction   

Fragments are small organic molecules, often with a molecular weight (MW) of below 200 Da, which can form weak but still specific interactions with proteins. While the low binding affinity of fragments cannot induce a reliable modulation of the activity of their target proteins, their simple structures have the potential to translate into high-efficiency recognition (Carr *et al.*, 2005 ▸). This renders fragments a good starting point for the development of ligands with much higher potencies. The past decade has witnessed an emerging interest in the application of fragments within drug-discovery projects (fragment-based lead discovery; FBLD), with four FBLD-guided drugs being approved for clinical usage to date (https://www.clinicaltrials.gov/) and numerous drug candidates being enrolled in clinical trials (Erlanson *et al.*, 2016 ▸).

Unlike traditional screening assays, which are usually biochemical or cell-based, fragment screening is often performed utilizing a variety of biophysical techniques. The requirement to detect weak recognition events between a protein and a ligand demands outstanding sensitivity of these methods (Renaud *et al.*, 2016 ▸; Erlanson *et al.*, 2016 ▸). In FBLD, the most common methods are nuclear magnetic resonance (NMR) spectroscopy, surface plasmon resonance (SPR) biosensor technology, thermal denaturation assays (TDA; also denoted FTSA, ThermoFluor *etc.*) and X-ray crystallography-based fragment screening (XFS) (Lamoree & Hubbard, 2017 ▸). Although XFS requires a crystallizable target with adequate diffraction quality and demands relatively large quantities of protein and access to sophisticated instrumentation (*i.e.* high-throughput beamlines at modern storage-ring facilities or efficient in-house X-ray sources), the three-dimensional structure of a protein–fragment complex is available directly after the first round of a screening campaign. Because of the substantial impact of structural information on the development of an FBLD-centred hit into a lead compound, XFS can be considered to be one of the most efficient fragment-screening techniques (Davies & Tickle, 2011 ▸; Renaud *et al.*, 2016 ▸), despite its relatively low throughput, associated costs and demands on access to large-scale infrastructure, for example electron storage-ring facilities.

The production of multiple protein crystals, both in their ‘apo’ form (here meaning with no artificially introduced ligands) and in fragment-bound states, is an essential step in an XFS campaign. Crystals of protein–ligand complexes can be generated in two ways: either by co-crystallizing the protein with ligands or by soaking pre-grown apo crystals in a solution of the ligand(s) (Müller, 2017 ▸). The latter procedure, despite a set of drawbacks (Ehrmann *et al.*, 2017 ▸), significantly increases the throughput of the screening campaign and allows a higher degree of automation in all stages of the experiment (Öster *et al.*, 2015 ▸). Extra care must be taken during the design of a fragment library. XFS-adopted compound collections, irrespective of whether they are focused on a particular class of targets or are universal, usually have members smaller than the typical fragments that are assayed using other biophysical techniques (Hall *et al.*, 2014 ▸; Keserű *et al.*, 2016 ▸). Additionally, owing to the nature of the crystal-soaking procedure and the low affinity of the potential hits, XFS fragments are required to be very soluble under the soaking conditions, which are often as high as 100 m*M* and even higher. A good fragment library is also designed with follow-up chemistry in mind, and thus the selected compounds should have good synthetic tractability (Keserű *et al.*, 2016 ▸), be easily accessible and, preferably, have an adequate pool of analogues for structure–activity relationship (SAR) exploration of hit compounds and their cores (Lamoree & Hubbard, 2017 ▸)

## 2. Platform details   

**Figure 1:**
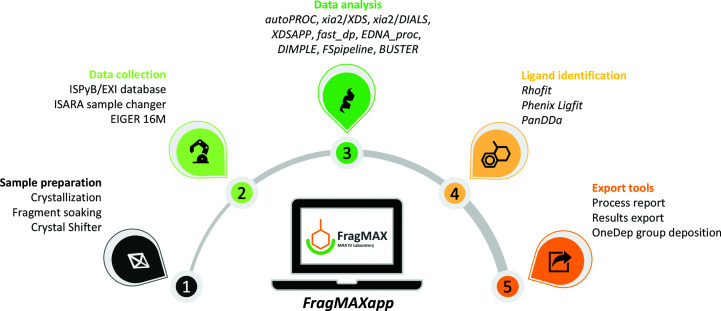
A typical experiment at FragMAX consists of five modules: (1) preparation of the crystals, manipulations with fragments, soaking of crystals and their harvesting, (2) data collection at the BioMAX beamline, (3) multiplex data analyses supported by the High-Performance Computing (HPC) infrastructure of the MAX IV Laboratory, (4) automated ligand-search methods and (5) export tools with support for the OneDep group deposition method. FragMAX projects are managed by our web application, *FragMAXapp*, which handles experimental information and results simply and intuitively.

FragMAX is a complete X-ray crystallography-based fragment-screening platform that utilizes the BioMAX macromolecular crystallography (MX) beamline (Ursby *et al.*, 2020 ▸) at the MAX IV Laboratory, Lund, Sweden. The platform benefits vastly from the extremely brilliant X-ray beam provided by the fourth-generation 3 GeV storage ring at the MAX IV Laboratory and the state-of-art instrumentation of the BioMAX beamline, with high-throughput data collection and automation of data processing. Although FragMAX provides optimal support for industrial users, it is also targeted at academic users. A typical FragMAX-assisted screening campaign, in which a protein with optimized crystallization and soaking conditions is screened against one or two entry-level 96-fragment libraries, takes around a week. To improve data analysis using *PanDDA* (Pearce *et al.*, 2017 ▸), users are advised to collect at least 40 apo structures during the experiment, making the initial campaign range from 136 to 232 data sets. During the experimental visit, which usually takes two to three days, training, sample preparation and data collection are performed. The downstream data analysis can be performed remotely, employing the in-house-developed web application *FragMAXapp*. A generalized pipeline for a fragment-screening experiment carried out at FragMAX is given in Fig. 1 ▸. Detailed information about access modes, open calls and user information can be found on the FragMAX webpage (https://www.maxiv.lu.se/fragmax).

Several fragment libraries are available to users, FragMAXlib, F2XEntry (Wollenhaupt *et al.*, 2020 ▸) and Xtal Frag Screen (Huschmann *et al.*, 2016 ▸), and academic users have free access to them. The platform is flexible and can easily adapt to external fragment libraries. If users wish to use their compounds, a CSV input file with SMILES notations of the structures and the corresponding fragment IDs must be provided. Additionally, the fragment library should be supplied in a platform-compatible format, as described in detail below. Data associated with fragment libraries will be processed with the *RDKit* (https://www.rdkit.org/) and *phenix.elbow* (Moriarty *et al.*, 2009 ▸) packages to generate 2D structural representations and the PDB and CIF files that are necessary for data analysis.

**Figure 2:**
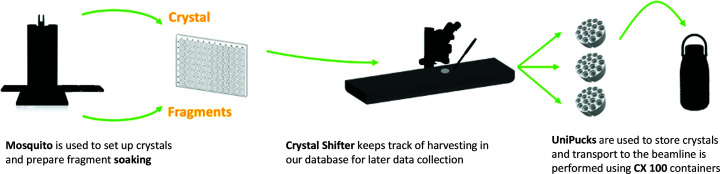
Sample-preparation overview. At FragMAX, crystallization plates are set up using the Mosquito with user-provided crystallization reagents and proteins. Crystal soaking can be performed using a liquid handler or manually, assisted by a Crystal Shifter instrument. Sample annotation is performed with the Crystal Shifter software together with *FragMAXapp*, which synchronizes with the ISPyB database for beamline operation.

Crystal soaking and other wet-laboratory manipulations take place at the associated facility, Lund Protein Production Platform [LP3; https://www.lu.se/lp3; https://portal.research.lu.se/portal/en/infrastructure/lund-protein-production-platform(c98f43d2-4907-496e-a0a8-6160645cf2fc).html], which is equipped with automated liquid-handling equipment. A Mosquito liquid-handling robot (TTP Labtech, UK) is used to prepare protein crystals and fragment plates, and to backfill fragment plates with the soaking solution. The platform is sensitive to the format of the plates, and currently supports MRC3 96-well plates (SWISSCI, Switzerland) and CrystalMation Intelli-Plates 96-3 low-profile (Rigaku, Japan). Soaking is usually performed by transfer of the diluted fragment and cryoprotectant to the crystallization drop, either manually with the assistance of a Crystal Shifter (Oxford Lab Technologies, UK), or optionally with a transfer protocol using the Mosquito robot. Even for the manual transfer, the current protocols allow the soaking of 96 fragments in 1 h. The concentration of fragment in the final mixture is defined by the amount of organic solvent (for example dimethyl sulfoxide, DMSO) that the crystals can sustain before losing their diffraction power. Crystal harvesting, annotation and definition of the experiment in the ISPyB database are also performed in a semi-automated manner using the Crystal Shifter (Fig. 2 ▸). Optionally, the experimental conditions (*i.e.* crystallization conditions, soaking concentration and cryoprotection) can be documented. This step is relevant for metadata-management purposes.

FragMAX provides the user with all necessary materials and equipment for on-site operations, such as crystallization plates, handling tools, MicroLoops LD of various sizes (MiTeGen, USA), UniPucks, shipping dewars and canisters. Additionally, parts of the equipment (for example UniPucks and dewars) can be lent to platform users.

**Figure 3:**
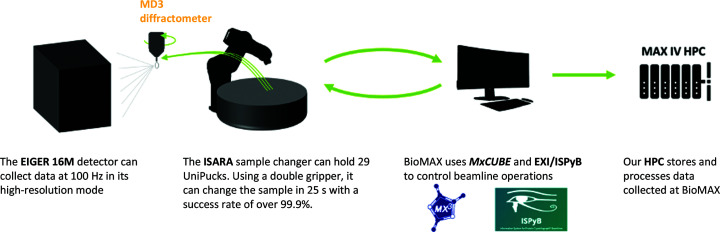
Data collection at BioMAX. FragMAX benefits from the modern and efficient infrastructure at the beamline. The ISARA sample changer supports large experiments (up to 464 crystals per single load), with fast sample changing and a very high success rate for crystal mounting. Experiments are controlled by the *MXCuBE*3 web interface and data processing is handled by software deployed at the MAX IV High-Performance Computing infrastructure.

Data collection takes place at BioMAX (Fig. 3 ▸), a state-of-the-art macromolecular crystallography beamline at the MAX IV Laboratory. BioMAX is tuneable in the energy range 5–25 keV, reaching 3 × 10^13^ photons per second × 0.05% bandwidth at 12.6 keV energy and 500 mA ring current. Users can select beam sizes ranging from 5 × 5 to 50 × 50 µm, which is ideal to match the loop or crystal size for optimal data collection. An ISARA sample changer (IRELEC, France) combined with a double-gripper tool, mounted on a six-axis robotic arm, facilitates screening projects with its capacity for 29 UniPucks (Crystal Positioning Systems, USA). The robotic arm allows fast (25 s for a full sample exchange) and reliable (less than 0.1% failure rate) sample manipulations (Ursby *et al.*, 2020 ▸). The operation is limited to sample-holder lengths (pin and cap) of 22 mm using a SPINE cap standard. The beamline operation can also be performed remotely by the user through an intuitive web-based version of *MXCuBE*3 (Mueller *et al.*, 2017 ▸).

The whole XFS experiment is underpinned from the very first step by a web application, *FragMAXapp*, which prepares ISPyB and *MXCuBE*3 for data collection and has minimal demands on the user’s side. *FragMAXapp* also allows users to have control over the data-analysis strategies. The data-processing run takes place at the MAX IV computing cluster, taking full advantage of its powerful parallelization setup. After data processing, *FragMAXapp* offers ample visualization, exploration and scoring tools, as explained further below. Additionally, the application arranges the results of the screening campaign in a format ready for database submission with group deposition systems, significantly reducing the time required for manual annotation of each model, its curation, validation and public release.

## 3. FragMAXlib   

**Figure 4:**
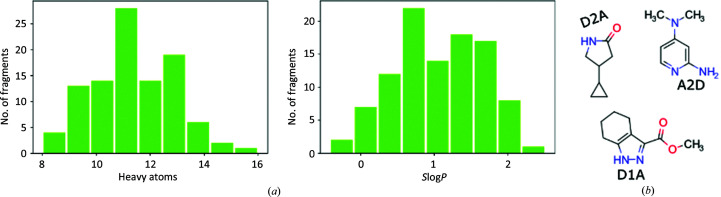
FragMAXlib: a fragment library for FragMAX. (*a*) Basic description of the library. The core set contains 96 compounds with an average size of 11 non-H (heavy) atoms. Predicted atom-based partition coefficients *S*log*P* were quantified according to Wildman & Crippen (1999 ▸). (*b*) Examples of library entries. Selection of the fragments was biased towards compounds with a close spatial arrangement of hydrogen-bond donors (D) and acceptors (A), *i.e.* with a topological pathway of one or two covalent bonds.

To encourage users who are new to crystallographic fragment screening, as well as to provide experienced users with additional screening options, FragMAX offers an XFS-focused fragment library. FragMAXlib contains a diverse set of 96 molecules, reflecting a common format for entry-level libraries (Huschmann *et al.*, 2016 ▸). The library is useful both for basic research and for validation of the ligandability of a target, supporting downstream FBLD activities. FragMAXlib entries were selected to be chemically inert and commercially available, with a maximum number of analogues present in commercial chemical space and accessible follow-up chemical transformations of hit compounds and analogues thereof (Taylor *et al.*, 2018 ▸; Keserű *et al.*, 2016 ▸; Erlanson *et al.*, 2016 ▸). The average size of a fragment in the first version of FragMAXlib is approximately 11 non-H atoms, with an average MW of 160 Da (Fig. 4 ▸ *a*). The library has minor deviations from Astex’s Rule of Three criterion (Ro3; Congreve *et al.*, 2003 ▸). Additionally, the compound-selection process was biased towards structures with minimal pharmacophore complexity and maximized polar pairs close to each other (Fig. 4 ▸ *b*), a practice hypothesized to improve the interaction of fragments with proteins (Keserű *et al.*, 2016 ▸). FragMAXlib is available in two formulations, with compounds solubilized either in DMSO or in ethylene glycol. The library is supplied in a platform-specific and single experiment-ready format and is dispensed in 0.15 µl aliquots to subwells of a crystallization plate. Detailed information about FragMAXlib and the other offered libraries can be found on the FragMAX project page (https://www.maxiv.lu.se/fragmax/fragmaxlib/).

## 4. User access   

FragMAX was designed to serve and be easily accessible to both industry and academia. The former can use proprietary access to MAX IV and retain tight control over their data and screening results. Academic users can access the platform services through a direct beamtime application during BioMAX open calls. Owing to the technical requirements needed to ensure a successful fragment-screening experiment and the relatively high associated costs, academic projects are pre-evaluated. The project evaluation includes tests for reproducible crystallization, crystal system stability under experimental conditions and the estimation of resolution limits during diffraction experiments.

When a new campaign starts, the users are offered training sessions for sample handling, project management and data processing using the FragMAX equipment and software. Attendance at the training is a mandatory requirement and must be undertaken by every participant in the experiment.

Industrial users will benefit from confidential data handling at all stages of the experiment from sample annotation to data analysis. The industrial user directly controls who can decrypt the results of a screen and all of the relevant materials. No outside party, including the BioMAX staff, has access to this information unless access is granted by the user. This is extremely advantageous for projects that involve pharmaceutical targets or undisclosed proprietary compounds.

## 5. FragMAX performance   

**Figure 5:**
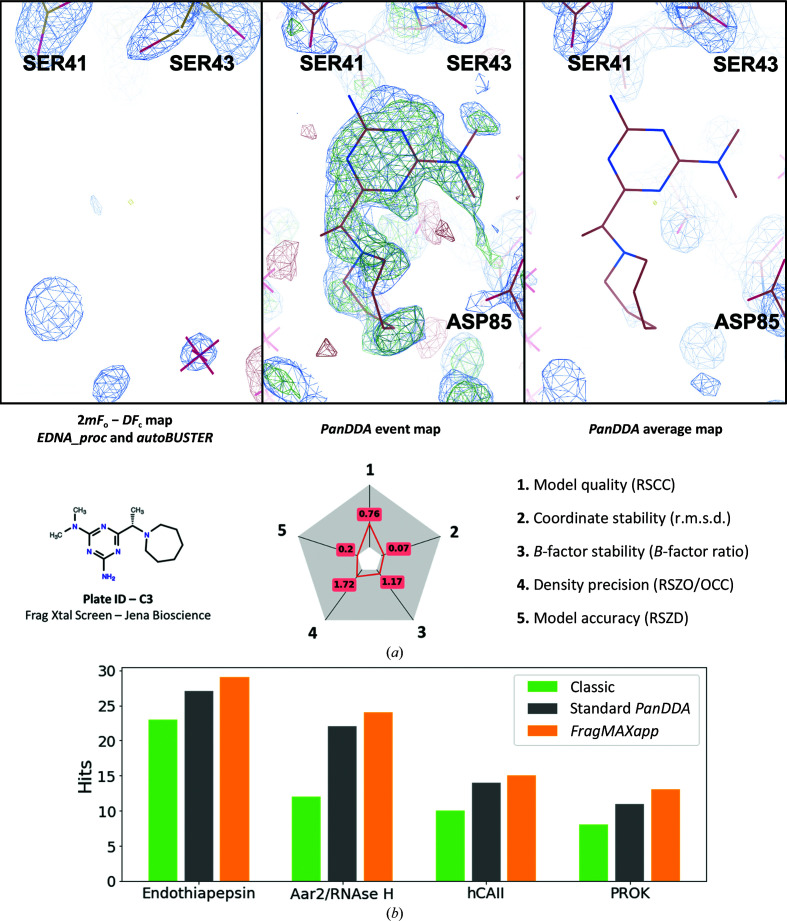
Evaluation of FragMAX performance. (*a*) Close-up view of the binding pocket for a hit found with *FragMAXapp* in a screening experiment with human carbonic anhydrase II. No ligand-related electron-density peaks were observed in a (2*m*|*F*_o_| − *D*|*F*_c_|) electron-density map from an autorefined method (*i.e.* direct output from *DIMPLE*, *FSpipeline* or *BUSTER*) contoured at 0.2973/1.1370 e Å^−3^ (1.50 r.m.s.d.). In contrast, *PanDDA* analysis and the corresponding event map revealed the ligand, and its relevance can be assessed by comparing the event map with the average map created with apo data sets. The figure shows the event map and the average map side by side, as available in *FragMAXapp*. Bottom: structure of the hit compound (left) and radial plot for the ligand-fit metrics (right) generated using the *PanDDA giant* scripts. (*b*) Comparison of the numbers of hits found with different proteins employing three different data-analysis approaches: a manual examination of unexplained density peaks found by *Coot* (‘Classic’, green bars), *PanDDA* analysis of a single combination of data-processing and structure-refinement pipelines (‘Standard *PanDDA*’, grey) and *PanDDA* analysis of the best solutions, defined by highest resolution, lowest *R* factors and highest ISa, from 18 possible combinations of data-analysis and structure-refinement pipelines offered in *FragMAXapp* (‘*FragMAXapp*’, orange). Endothiapepsin (hit rate = 30%) and Aar/RNaseH (hit rate = 21%) are user project targets; hCAII, human carbonic anhydrase II (hit rate = 15%); PROK, proteinase K (hit rate = 13%). The described hit rate is based on *FragMAXapp* analysis.

FragMAX data-analysis solutions rely on *PanDDA* analysis. Following *PanDDA*, higher hit rates and higher sensitivity of the fragment screening can be achieved. To exemplify, human carbonic anhydrase II was crystallized as described in Fisher *et al.* (2009 ▸) and submitted to FragMAX for screening against the Xtal Frag Screen library (Jena Bioscience, Germany; Huschmann *et al.*, 2016 ▸). A comparison between different approaches to search for ligands in electron densities highlights the way that FragMAX benefits from the modern data-processing methods that are available. In Fig. 5 ▸(*a*), searching for unexplained density, here called the ‘Classic’ method (green bars in the chart in Fig. 5 ▸ *b*), leads to water placement in spherical-shaped electron-density peaks, whilst the multi-data-set analysis provided by *PanDDA* (grey bars in the chart in Fig. 5 ▸ *b*) can more clearly distinguish a ligand bound to the protein near Ser43, Asp139, Lys127 and Asp85. The right-hand side of Fig. 5 ▸(*a*) shows the ground-state map, which is useful for removing bias when placing ligands in the event map.

Additionally, the platform offers an improved *PanDDA*-based data-analysis workflow, denoted *FragMAXapp* in Fig. 5 ▸(*b*). It classifies outputs from multiple combinations of various automated pipelines, selects the optimal output for each unique crystal by ranking the best resolution, *R*_work_/*R*_free_, ISa and *R*_merge_, and submits the selection output for *PanDDA* analysis. At the time of this publication, five independent user projects have been performed with four different proteins. Initial evaluation of the platform performance demonstrated that *FragMAXapp*-assisted multiplex data analysis led to higher hit ratios than the classic methods (Fig. 5 ▸ *b*). In the multiplex strategy, a sequence of automated pipelines for data processing, structure solution and ligand fitting are combined, resulting in up to 36 outputs for each data set. For the tested proteins, a multiplex approach to data processing proved to be efficient, finding more screening hits than conventional methods. Here, a hit is defined as a unique interaction event with the target. If the same fragment is observed in two different binding sites, they are described as two hits as they could lead to two independent fragment-optimization routes. The *FragMAXapp* method will be described and exemplified in detail elsewhere (Lima *et al.*, in preparation). The proteins used for comparing the different data-analysis strategies are human carbonic anhydrase II (hCAII) and proteinase K (PROK) screened against the Xtal Frag Screen (Jena Bioscience, Germany), with 96 fragments dissolved in DSMO, and endothiapepsin (EP) and Aar/RNaseH (AR) screened against the F2X Entry library (Wollenhaupt *et al.*, 2020 ▸), with 96 fragments dissolved in DMSO.

## 6. Conclusions   

Taking advantage of modern and robust data-collection and data-processing methods, macromolecular crystallography allows the medium-throughput screening of fragments. The contemporary state of XFS can satisfy the needs of a broad range of research and development projects, both industrial and academic. With improved data-analysis tools and the ability to collect data sets from several hundred crystals in a short period of time using brilliant X-ray sources, the first synchrotron-based fragment-screening facilities are showing success. MAX IV FragMAX, together with currently running facilities such as XChem at Diamond Light Source, Didcot, UK (Krojer *et al.*, 2017 ▸), Helmholtz-Zentrum Berlin, Berlin, Germany (https://www.helmholtz-berlin.de/forschung/oe/np/gmx/fragment-screening/index_en.html) and CrystalDirect at ESRF, France (Cipriani *et al.*, 2012 ▸), is expected to contribute significantly to the FBLD field. Recently, the devastating outbreak of COVID-19 (Wu *et al.*, 2020 ▸) has highlighted how high-throughput and automated methods are important for a rapid response to disease, as exemplified by the work performed by the XChem group at Diamond Light Source, who screened several libraries against the SARS-CoV-2 main protease M^pro^ (Günther *et al.*, 2020 ▸; Douangamath *et al.*, 2020 ▸). FragMAX widens the available choice of facilities for XFS that will be at the disposal of users to explore links between the structures and functions of proteins and the finest details of molecular recognition between proteins and their ligands. Thereby, the new platform makes the methodology of fragment screening more accessible to the user community internationally.
